# Reproducibility of the energy metabolism response to an oral glucose tolerance test: influence of a postcalorimetric correction procedure

**DOI:** 10.1007/s00394-022-02986-w

**Published:** 2022-08-25

**Authors:** Juan M. A. Alcantara, Guillermo Sanchez-Delgado, Lucas Jurado-Fasoli, Jose E. Galgani, Idoia Labayen, Jonatan R. Ruiz

**Affiliations:** 1 Department of Physical and Sports Education, Faculty of Sport Sciences, PROFITH “PROmoting FITness and Health Through Physical Activity” Research Group, Sport and Health University Research Institute (iMUDS), University of Granada, 18011 Granada, Spain; 2 Pennington Biomedical Research Center, Baton Rouge, LA 70808 USA; 3 Department of Physiology. Faculty of Medicine, University of Granada, Av. Conocimiento s/n, 18011 Granada, Spain; 4 Department of Health Sciences, Nutrition and Dietetics Career, Faculty of Medicine, Pontificia Universidad Católica de Chile, Santiago, Chile; 5 Department of Nutrition, Diabetes and Metabolism, Faculty of Medicine, Pontificia Universidad Católica de Chile, Santiago, Chile; 6 Institute for Innovation & Sustainable Food Chain Development, Department of Health Sciences, Public University of Navarra, Campus Arrosadía, s/n., Pamplona 31006, Spain; 7 Instituto de Investigación Biosanitaria, Ibs.Granada, Granada, Spain

**Keywords:** Indirect calorimetry, Reliability, Postprandial metabolism, Glucose tolerance, Metabolic cart

## Abstract

**Purpose:**

Metabolic flexibility (MetF), which is a surrogate of metabolic health, can be assessed by the change in the respiratory exchange ratio (RER) in response to an oral glucose tolerance test (OGTT). We aimed to determine the day-to-day reproducibility of the energy expenditure (EE) and RER response to an OGTT, and whether a simulation-based postcalorimetric correction of metabolic cart readouts improves day-to-day reproducibility.

**Methods:**

The EE was assessed (12 young adults, 6 women, 27 ± 2 years old) using an Omnical metabolic cart (Maastricht Instruments, Maastricht, The Netherlands) after an overnight fast (12 h) and after a 75-g oral glucose dose on 2 separate days (48 h). On both days, we assessed EE in 7 periods (one 30-min baseline and six 15-min postprandial). The ICcE was performed immediately after each recording period, and capillary glucose concentration (using a digital glucometer) was determined.

**Results:**

We observed a high day-to-day reproducibility for the assessed RER (coefficients of variation [CV] < 4%) and EE (CVs < 9%) in the 7 different periods. In contrast, the RER and EE areas under the curve showed a low day-to-day reproducibility (CV = 22% and 56%, respectively). Contrary to our expectations, the postcalorimetric correction procedure did not influence the day-to-day reproducibility of the energy metabolism response, possibly because the Omnical’s accuracy was ~ 100%.

**Conclusion:**

Our study demonstrates that the energy metabolism response to an OGTT is poorly reproducible (CVs > 20%) even using a very accurate metabolic cart. Furthermore, the postcalorimetric correction procedure did not influence the day-to-day reproducibility.

*Trial registration* NCT04320433; March 25, 2020.

**Supplementary Information:**

The online version contains supplementary material available at 10.1007/s00394-022-02986-w.

## Introduction

The assessment of whole-body energy metabolism using the indirect calorimetry (IC) technique is a widely used tool to better understand the energy homeostasis in humans [1]. Nowadays, IC is a versatile tool that offers a wide range of applications and is commonly used to determine the influence of nutrients (e.g., metabolic flexibility), drugs, biocompounds, etc. on thermogenesis and fuel oxidation metabolism. As abovementioned, IC can be used to assess the metabolic flexibility (MetF)—which refers to the capacity to adapt fuel oxidation to fuel availability and energy demand [2]. From a whole-body perspective, MetF is widely considered a surrogate of metabolic health [2]. MetF is related to energy balance and energy intake regulation, and it has been suggested that an impaired MetF is associated with an increased risk of body weight gain and metabolic disorders [3–9].

Nowadays, the most used procedure for assessing MetF is the euglycemic–hyperinsulinemic clamp [10, 11]. However, this procedure is not generally accessible for being used in epidemiologic or large-cohort studies [12]. Thus, other procedures such as the oral glucose tolerance test (OGTT) [13] that employs easier laboratory tests and protocols are increasing their popularity [12]. When assessing MetF by an OGTT, the postprandial oxygen consumption (VO_2_) and carbon dioxide production (VCO_2_) can be measured using a metabolic cart, and the respiratory exchange ratio (RER) estimated [14–18]. Then, MetF is commonly determined as the increase in RER during an OGTT [2, 16]. Unfortunately, many metabolic carts have shown limited accuracy and a low day-to-day reproducibility [19–24]. That could directly influence the gas exchange measurements preventing an accurate determination of RER changes during the postprandial gas exchange assessment [16].

In an attempt to enhance the accuracy and day-to-day reproducibility of the metabolic carts, Schadewaldt et al. [25] proposed a procedure to correct the metabolic carts readouts using controlled pure nitrogen (N_2_) and carbon dioxide (CO_2_) concomitant gas infusions (hereinafter *individual calibration control evaluation* [ICcE]). In brief, after the participant’s gas exchange recording, the ICcE is carried out to simulate VO_2_ and VCO_2_ rates, allowing the determination of the metabolic cart error. Later, this error is used to correct the participant’s indirect calorimetry readouts [25]. Galgani et al. [16] used the ICcE procedure after two consecutive OGTTs (ingested 3-h apart), and observed that the RER pattern yielded by the “corrected” data better represented the expected physiological response compared to the “uncorrected” metabolic cart data [16]. Thus, they suggested that the application of the ICcE procedure could improve the postprandial assessments [16]. Therefore, it is conceivable that the ICcE procedure increases the day-to-day reproducibility of postprandial gas exchange (i.e., increases the RER reproducibility) [26], although it has not been tested yet.

In this study, we determined the day-to-day reproducibility of gas exchange before and after an OGTT in young healthy adults from two non-consecutive (48-h apart) tests. Moreover, we also aimed to determine whether the ICcE proposed by Schadewaldt et al. [25] influences the day-to-day reproducibility of the measured gas exchange parameters.

## Methods

### Subjects

Twelve young adults (6 men, 6 women) participated in the present study. Of note, we did not conduct *a priory* sample size calculation. The inclusion criteria were: (i) being older than 18 years old; (ii) having a body mass index (BMI) between 18.5 and 27.5 kg/m^2^ (inclusive); (iii) having a stable body weight over the last 3 months (i.e., changes < 3 kg) and not being enrolled in a weight loss program during the study; (iv) being non-smokers; (v) not being under medication that could directly influence energy metabolism; (vi) not suffering from chronic (e.g., impaired glucose metabolism, diabetes) or acute illness; and (vii) not being pregnant or lactating.

All previous inclusion criteria were verbally confirmed by all the participants. The study protocol and written informed consent followed the 2013 revised Declaration of Helsinki and was approved by the Human Research Ethics Committee of the University of Granada (1102/CEIH/2020). The study was registered in Clinicaltrials.gov (NCT04320433).

### Experimental design

#### Study visits

**Fig. 1:**
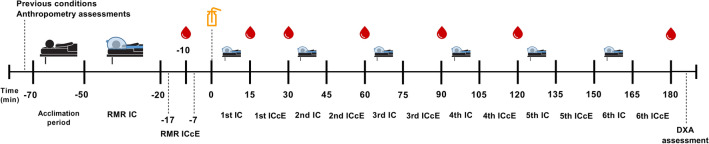
Study design (replicated on both testing days, 48 h apart). The anthropometry assessments included height and weight. IC: indirect calorimetry (using a metabolic cart) assessments. RMR: resting metabolic rate assessment/period; ICcE: individual calibration control evaluation proposed by Schadewaldt et al. [25]. The bottle icon represents the 75-g oral glucose dose intake. The drop icons represent the capillary blood glucose level assessments. DXA: dual-energy X-ray absorptiometry assessment. Anthropometry and DXA assessments were performed only on day 1. The study protocol timeline is expressed as minutes (min)

The study design is presented in Fig. 1. Gas exchange was measured in fasting (12 h; hereinafter resting metabolic rate [RMR] period) conditions and after a 75-g oral glucose dose (NUTER TEC: orange flavor, Toulouse, France) using the Omnical metabolic cart system (Maastricht Instruments, Maastricht, The Netherlands). Tests were performed on 2 non-consecutive days (48 h apart) and started in the morning (≈9 am). The participant’s gas exchange was collected equipping the Omnical system with a plastic canopy-hood. Of note, the flow and the gas analyzers of the Omnical system were automatically calibrated accordingly to the manufacturer’s instructions each testing day and prior the gas exchange measurement.

Participants arrived at the research center by motorized vehicle avoiding any moderate or intense physical activity, since they woke up. Furthermore, participants refrained from vigorous and moderate intensities of physical activity during the preceding 48 h and 24 h, respectively. On the testing day, participants confirmed having consumed the standardized 24-h *ad-libitum* meal plan (Table S1), including the dinner 12-h before the start of the baseline assessment (i.e., the RMR period) to ensure that they met the 12-h fasting period. From the two options (Table S1), participants were instructed to select and follow one (i.e., the menu was not exchangeable). Prior the first visit, participants had to register the amount they ate in a food diary to replicate food intake. Moreover, they avoided consuming any stimulant beverages (e.g., coffee and tea) or drugs that could influence metabolism during the preceding 24-h period. All conditions were replicated prior the second testing day.

On both days, we measured the gas exchange in 7 periods, one 30-min fasting (RMR period) and six 15-min postprandial gas exchange measurements (Fig. 1). The ICcE procedure proposed by Schadewaldt et al. [25] was performed after each gas exchange measurement (Fig. 1). The metabolic cart recording was not interrupted during the whole test. The gas exchange measurement was performed in agreement with the current indirect calorimetry testing recommendations [27]. Before the RMR period, participants rested motionless on a reclined bed in the supine position for 20 min. Furthermore, they were also asked to lay on the bed for 10 min before each postprandial gas exchange measurement (Fig. 1). Participants were instructed to breathe normally, and not to sleep, talk or fidget during the gas exchange measurements [27]. Regarding the room conditions, all the tests were carried out in the same dim lighting and quiet room, under controlled ambient temperature and humidity (22.5 ± 0.7ºC and 22.6 ± 0.4ºC, and 36.8 ± 6.6% and 35.4 ± 6.4% for Day 1 and Day 2, respectively).

The oral glucose drink was offered, and participants had 2 min for ingesting it (Fig. 1). The glucose intake was performed under the metabolic cart’s canopy using a plastic straw, while the participants were in a semireclined position to consume the beverage. Capillary blood samples were obtained 10 min prior to the glucose ingestion and 15, 30, 60, 90, 120 and 180 min after its ingestion (Fig. 1) by a finger stick using a lancet (sterile lancet Acofar, Acofarma, Terrasa, Barcelona). Then, capillary glucose concentration (hereinafter *glucose concentration*) was determined using a digital glucometer (Contour® XT Blood Glucose Meter, Bayer, Basel, Switzerland) equipped with a blood glucose test strip (Contour® XT Blood Glucose Test Strips, Bayer, Basel, Switzerland).

Every week (the study lasted 9 weeks in total), we performed routine methanol burnings and controlled N_2_ and CO_2_ pure gas infusions (see **b** for detailed information) to test the accuracy of the metabolic cart.

#### Individual Calibration control Evaluation procedure (ICcE)

Immediately after each participant’s gas exchange measurement using the Omnical metabolic cart (representing the *participant’s readout* values), pure N_2_ and CO_2_ (Carburos Metálicos/Air Products and Chemicals, Inc., Barcelona, Spain; purities ≥ 99.9997% and ≥ 99.995%, respectively) were directly and concomitantly infused during 10 min into the Omnical’s hose tube at fixed flows of 1.115 and 0.25 L per minute, respectively [25]. To infuse both gases, two high-precision mass-flow controllers (358 Series, Analyt-MTC, Müllheim, Germany; flow range: 0–2 l/min) were used, one for each gas. The infused gases are used to calculate the *expected values* (i.e., these gases used to simulate VO_2_ and VCO_2_ values). Later, the Omnical’s VO_2_ and VCO_2_ readouts during the last 5 min of the infusion were averaged, representing the *measured values*. Therefore, the *corrected values* for VO_2_ and VCO_2_ were calculated as presented below

1 $$Corrected \,value = \frac{{participant^{\prime}s \,readout \times expected \, value }}{measured \,value}$$

Of note, we considered that the infused pure CO_2_ is equivalent to the VCO_2_ [25]. The simulated VO_2_ (as the pure N_2_ was used to dilute the O_2_ present in ambient air) can be calculated as presented in Eq. 2 [28﻿]

2 $$VO_{2}  \;\left( {ml/min} \right) = infused \,\, N_{2} \;\left( {ml/min} \right) \times 0.2646$$

#### Gas exchange parameters’ calculation

The VO_2_ and VCO_2_ data were downloaded from the Omnical metabolic cart every 5 s. For the RMR period, both the first and last 5-min data were discarded and the remaining 20-min data were averaged. Regarding the postprandial data (for each measured period), the first 5-min data were discarded and the remaining 10 min averaged. Then, the RER (i.e., VCO_2_-to-VO_2_ ratio) was calculated, and the abbreviated Weir equation [29] used to estimate the RMR and the postprandial energy expenditure (EE). Carbohydrate (CHO) utilization was estimated using the Frayn’s equation [30]. For RMR, EE, and CHO utilization, the nitrogen urinary excretion was considered to be 0. Finally, to compute in a single outcome the postprandial measured gas exchange response, we calculated the incremental area under the curve (AUC) by the trapezoidal rule [31] minus the baseline (i.e., RMR period) value for each parameter (VO_2_, VCO_2_, RER, EE, and CHO utilization). Importantly, the same calculations were performed with the *uncorrected* and the *corrected* (i.e., after performing the ICcE procedure), as well as for the glucose concentration values.

#### Anthropometric and body composition assessments

On the first testing day (Fig. 1), a stadiometer and scale were used to measure height and weight (Seca model 799, Electronic Column Scale, Hamburg, Germany), while participants wore light clothing and no shoes. Then, the body mass index (BMI) was calculated as the weight (in kilograms) divided by the squared height (in meters). At the end of the first testing day, body composition (lean mass, fat mass, and fat mass percentage) was determined using a whole-body dual-energy X-ray absorptiometry scanner (Discovery Wi, Hologic, Inc., Bedford, MA, USA).

#### Routine metabolic cart accuracy testing

Weekly (9 weeks in total) and prior to the first testing day, we performed methanol burning and pure gas infusions tests to ensure that the metabolic cart performance was optimal for its intended use. Both, gases analyzers and flow sensor calibrations were performed daily in accordance with the manufacturer’s recommendations.

We calculated the measurement error as the following example:

$$VO_{2} \;measurement\; error\; \left( {ml/min} \right) = measured \;VO_{2} \; value \;\left( {ml/min} \right) - expected\; VO_{2} \; value \;\left( {ml/min} \right),$$

where the *measured value* is the metabolic cart readout and the *expected value* is the theoretical value expected to be retrieved by either the methanol burning or the pure gas infusions.

#### Methanol burning tests

All burning tests were performed by the same researcher (JMAA). The methanol burning test procedure was as follows: first, pure (water ≤ 0.05%, purity ≥ 99.9%) methanol (EMSURE® ACS, ISO, Reag. Ph Eur, Merck, Darmstadt, Germany) was placed inside the methanol burning cage (Maastricht Instruments, Maastricht, The Netherlands) and the flame of the methanol burning kit was lighted. Then, the gases produced by the combustion were directed to the metabolic cart hose tube, while the combusted methanol weight was dynamically recorded using a high-precision scale (model MS 1602TS/00 precision scale, precision 0.01 g; Mettler Toledo, Giessen, Germany). The expected value for the RER was 0.667 [21, 32], while the expected VO_2_ and VCO_2_ recovery values were 100% (i.e., 100% recovery = 0% measurement error). From the whole gas exchange data recording (the minimum duration of each test was 25-min), the first 5 min of data were retrospectively discarded, and the remaining data averaged for analyses.

#### Gas infusion tests

The duration of each controlled pure gas infusion test was 15 min, and all the tests were performed by the same researcher (JMAA). The infusions tests were performed following exactly the same procedures that we did for the *ICcE procedure* described above. As for the burning tests, the expected VO_2_ and VCO_2_ recoveries values were 100%, and the first 5 min of data were retrospectively discarded for ensuring a proper gas exchange mixture and the remaining data averaged for analyses.

### Statistical analyses

Results are presented as mean ± standard deviation (SD), unless otherwise stated. All figures were created using the Graph Pad Prism software (GraphPad, v. 8.0.2, CA, USA), while analyses were performed using the Statistical Package for Social Sciences v. 22 (SPSS, IBM SPSS Statistics, IBM Corporation, Chicago, IL, USA). The level of significance was set at *P* value ≤ 0.05.

A two-factor (Time × *ICcE* [i.e., uncorrected; corrected]) ANOVA, with LSD Tukey comparisons, was used for each visit separately. Then, to test between-day differences in RER, EE, VO_2_, VCO_2_, and CHO utilization, and AUCs, a two-factor ANOVA (Visit [i.e., Day 1; Day 2] × IC*cE*), with LSD Tukey comparisons, was used. On the other hand, for each time period and gas exchange parameter (i.e., corrected and uncorrected RER, EE, VO_2_, VCO_2_, and CHO utilization, and their respective AUCs), we calculated the day-to-day coefficient of variation (CV) as (SD / average) × 100. Additionally, to further study the day-to-day reproducibility of the gas exchange parameters, we computed the mean difference (Day 1 minus Day 2; also known as *mean bias*) and the 95% lower and upper limits of agreement (LoA) [33].

To test differences in mean glucose concentration obtained on both testing days, a two-factor (Time × Visit [Day 1; Day 2]) ANOVA, with LSD Tukey comparisons, was used. Furthermore, to test between-day differences in AUC glucose concentration, a paired t test was used. Then, the CV was calculated for the glucose concentration values obtained at each time period. Finally, for glucose concentration, we also computed the mean difference and the 95% lower and upper LoA as abovementioned [33].

## Results

A total of 6 men (28 ± 2 years old, 176 ± 3 cm, 76 ± 7 kg, 57 ± 4 kg lean mass, and 20 ± 6% fat mass) and 6 women (26 ± 2 years old, 164 ± 5 cm, 60 ± 4 kg, 34 ± 3 kg lean mass, and 38 ± 4% fat mass) participated in the study. None of them reported any adverse event after the 75-g oral glucose dose intake. The weekly (*n* = 9) methanol burns and controlled pure gas infusion tests showed, on average, that the accuracy of the Omnical system was ≈100% (Figure S1).

**Fig. 2:**
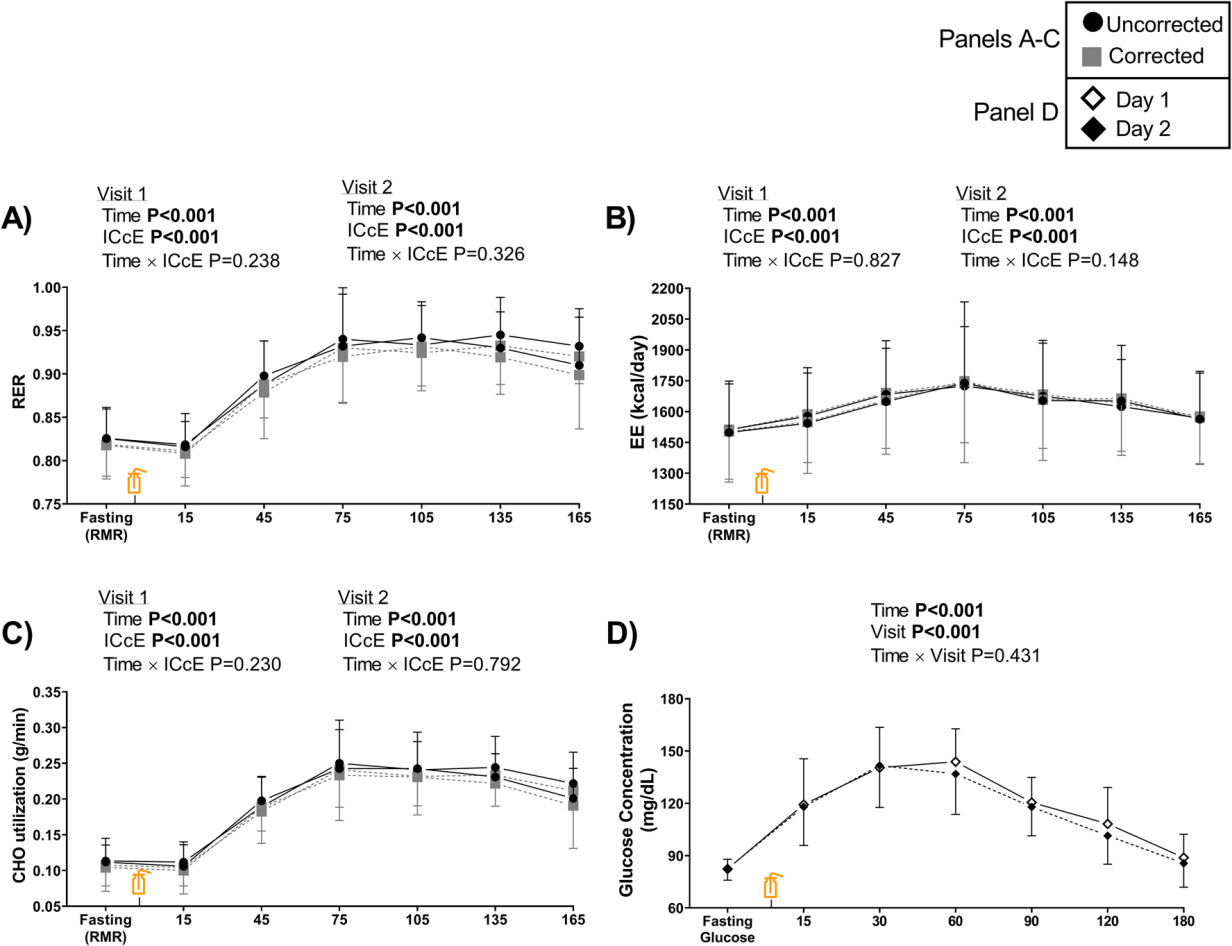
Respiratory exchange ratio (RER, **A**), energy expenditure (EE, **B**), and carbohydrate (CHO) utilization (**C**) with and without applying the individual calibration control evaluation procedure (ICcE), and capillary blood glucose concentrations (**D**) obtained on Visit 1 and Visit 2 tests. Fasting RMR values correspond to the resting metabolic rate (RMR) period, i.e., before the glucose intake, while 15, 45, 75, 105, 135, and 165 represent the time in minutes for gas exchange data after the glucose intake. The bottle icon (*x*-axis) represents the moment in which the glucose (75-g dose) was provided. P values from two-factor (Time × ICcE) repeated-measures analysis of variance (ANOVA, *n* = 12, **A**-**C**) for Day 1 and Day 2 comparisons. P values from two-factor (Time × Visit) ANOVA (*n* = 12, Panel D). Results are presented as mean and standard deviation

On both testing days, no *Time* × *ICcE* interaction effect was observed on RER (Fig. 2A) or EE (Fig. 2B). Interestingly, the RER and EE patterns were similar regardless of the correction of the gas exchange data (Fig. 2). On both testing days and regardless of the ICcE procedure, *post hoc* analyses showed RER differences between the RMR IC time period (i.e., fasting RMR) compared to the 2nd IC (45-min), 3rd IC (75-min), 4th IC (105-min), 5th IC (135-min), and 6th IC (165-min) periods (all *P* < 0.001), while for EE differences were observed vs. the 2nd, 3rd, 4th and 5th IC periods (all *P* ≤ 0.033). The uncorrected and corrected AUC RER and AUC EE values were similar on both visits (all *P* ≥ 0.688; Table S2). The results for uncorrected and corrected VO_2_ and VCO_2_ (Figure S2) were similar to these aforementioned for RER and EE (Fig. 2). Additionally, uncorrected and corrected AUC VO_2_ and AUC VCO_2_ are presented in Table S2. Uncorrected and corrected AUC values for the above-mentioned parameters were similar, although an interaction effect (Visit × ICcE) on AUC EE and AUC VO_2_ was observed (both P < 0.05; Table S2). On both testing days, no Time × ICcE interaction effect was observed on CHO utilization (Fig. 2C), and *post hoc* analyses showed CHO utilization differences similar to these observed for RER (Fig. 2A). Uncorrected and corrected AUC CHO utilization values were similar on both visits (both *P* ≥ 0.660; Table S2).

**Fig. 3:**
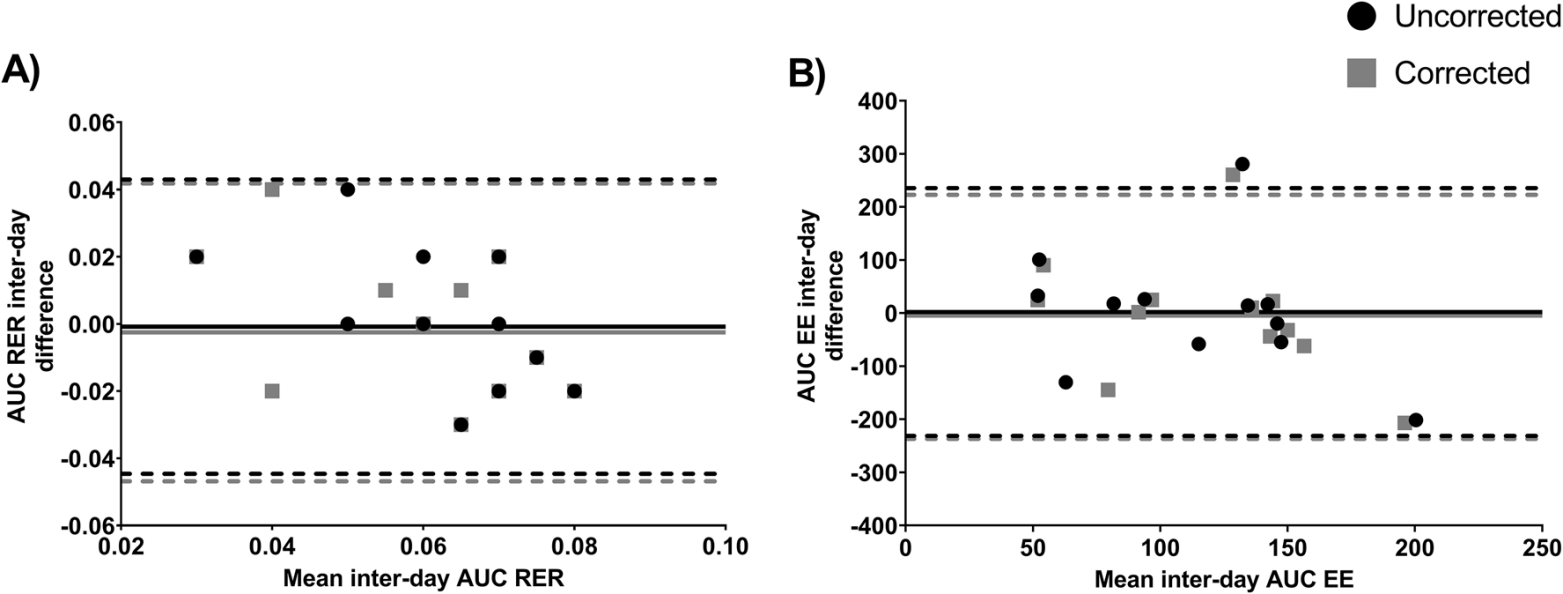
Bland–Altman plots for inter-day reproducibility of the area under the curve for the respiratory exchange ratio (AUC RER; **A**) and for the energy expenditure (AUC EE; **B**), with and without applying the individual calibration control evaluation procedure (*n* = 12). Solid line represents the bias (systematic error) between day 1 and day 2. Dashed lines represent the upper and the lower limits of agreement (mean ± 1.96 standard deviation)

Table 1 shows the day-to-day reproducibility of RER and EE parameters. Overall, mean differences, lower and upper LoA, and CV were similar between uncorrected and corrected values for both RER and EE (Table 1). Mean differences and LoA were also similar between uncorrected and corrected values of the AUC RER (Fig. 3A) and the AUC EE (Fig. 3B). The CV for uncorrected and corrected AUC RER were 22 ± 18% and 26 ± 19%, while for the AUC EE values were 56 ± 56% and 50 ± 52%, respectively. On the other hand, the day-to-day reproducibility of uncorrected and corrected VO_2_, VCO_2_ and CHO utilization and their respective AUCs is presented in Table S3. Overall, we observed similar results for uncorrected and corrected values, and the CV for uncorrected and corrected AUCs were high (all CVs > 23%; Table S3).

**Table 1:** Day-to-day reproducibility and coefficient of variation (CV) for respiratory exchange ratio (RER) and energy expenditure (EE) with (corrected) and without (uncorrected) applying the individual calibration control evaluation procedure parameters at each time period

	Uncorrected values			Corrected values		
	Mean difference (SD)	95% LoA (lower; upper)	CV	Mean bias (SD)	95% LoA (lower; upper)	CV
**Time period**						
RMR IC						
RER	0.01 (0.03)	(− 0.06; 0.06)	2.0 (1.3)	0.01 (0.03)	(− 0.06; 0.06)	2.2 (1.4)
EE (kcal/day)	− 14 (117)	(− 243; 216)	4.0 (2.9)	− 9 (117)	(− 238; 220)	3.9 (3.0)
1st IC						
RER	0.01 (0.03)	(− 0.07; 0.06)	1.9 (2.2)	0.01 (0.03)	(− 0.07; 0.06)	1.9 (2.3)
EE (kcal/day)	− 35 (123)	(− 276; 207)	4.2 (3.3)	− 37 (126)	(− 284; 210)	4.4 (3.4)
2nd IC						
RER	0.01 (0.04)	(− 0.07; 0.09)	2.8 (1.6)	0.01 (0.04)	(− 0.07; 0.09)	2.6 (1.8)
EE (kcal/day)	− 35 (63)	(− 159; 90)	2.3 (1.6)	− 35 (66)	(− 165; 95)	2.2 (1.9)
3rd IC						
RER	− 0.01 (0.05)	(− 0.11; 0.09)	3.2 (2.2)	− 0.01 (0.05)	(− 0.11; 0.09)	3.1 (2.3)
EE (kcal/day)	15 (273)	(− 525; 555)	8.5 (5.7)	10 (280)	(− 538; 558)	8.7 (5.7)
4th IC						
RER	0.01 (0.06)	(− 0.11; 0.12)	3.6 (2.4)	0.01 (0.05)	(− 0.10; 0.12)	3.4 (2.3)
EE (kcal/day)	− 22 (105)	(− 228; 184)	3.6 (2.6)	− 25 (98)	(− 216; 166)	3.5 (2.5)
5th IC						
RER	− 0.02 (0.04)	(− 0.10; 0.07)	2.6 (2.1)	− 0.01 (0.04)	(− 0.09; 0.06)	2.5 (1.8)
EE (kcal/day)	28 (108)	(− 183; 239)	3.4 (2.8)	23 (114)	(− 201; 246)	3.6 (2.8)
6th IC						
RER	− 0.02 (0.06)	(− 0.13; 0.09)	3.7 (2.7)	− 0.02 (0.05)	(− 0.12; 0.08)	3.6 (2.4)
EE (kcal/day)	− 5 (82)	(− 167; 156)	3.0 (2.0)	− 5 (84)	(− 169; 159)	2.8 (2.4)

Results are presented as mean difference (day 1 minus day 2) and standard deviation (SD), 95% limits of agreement (LoA; lower and upper limits), and CV expressed as percentage and (SD). RMR IC is the resting metabolic rate period. Indirect calorimetry (IC) 1st to 6th denotes the period in which the gas exchange was recorded

For all participants, fasting glucose concentrations values were normal (82 ± 6 mg/dl and 83 ± 7 mg/dl for day 1 and day 2 respectively). No Time × Visit interaction effect on glucose concentration was observed (Fig. 2D). We observed that on both testing days, the glucose concentration at the end of the test was similar to the fasting levels, and no post hoc differences (both *P* values ≥ 0.147) were observed. As expected, we observed higher RER values (Fig. 2A) as glucose concentrations increased (Fig. 2D). The AUC glucose concentration values were 0.06 ± 0.01 and 0.06 ± 0.02 ml/dl × min for day 1 and day 2, respectively (*P* = 0.090). Table S4 shows the day-to-day reproducibility of glucose concentration. Overall, although the mean differences were low for all time periods, we observed wide LoA (Table S4).

## Discussion

The present study determined the day-to-day reproducibility of gas exchange (RER, EE, VO_2_, and VCO_2_) in response to an OGTT in young healthy adults using the Omnical metabolic cart. When studying the day-to-day reproducibility of the measured gas exchange values during the different periods of the study, we observed low CV values (and narrow LoAs) for RER and EE, respectively. However, after computing the postprandial measured gas exchange response in a single outcome (i.e., as an AUC), we observed high CV values (and wide LoAs) for AUC RER and AUC EE. Thus, although the day-to-day reproducibility of the measured gas exchange values during each period (e.g., RMR period, 1^st^ IC period) is reproducible between days (all CVs < 10%, Table 1), after computing the entire gas exchange response in a single parameter (e.g., AUC RER), we did not observe the same trend, and the day-to-day reproducibility was lost (CV > 20%). Contrary to our hypothesis, the ICcE procedure proposed by Schadewaldt et al. [25] did not influence the day-to-day reproducibility of the postprandial gas exchange as determined by the Omnical metabolic cart. Moreover, the gas exchange parameters (e.g., RER and EE) for each period and the gas exchange parameters computed as a single outcome (e.g., AUC RER) were not modified by the ICcE procedure.

In a previous study, Galgani et al. [16] tested the influence of the ICcE procedure on postprandial gas exchange data (after two consecutive OGTTs performed the same day; 3-h apart), and the results were promising. In fact, they observed a different RER pattern between the uncorrected and the corrected values, being the later the “expected” (theoretical) pattern after the two glucose loads intake. Conversely, in our study (after a single OGTT), we observed the same pattern between the uncorrected and the corrected RER values. Importantly, the similar patterns we observed for uncorrected and corrected values after applying the ICcE procedure (Fig. 2, and Figure S2) could be explained by the high accuracy of the metabolic cart (Figure S1). In this regard, the measurement error observed for the Omnical metabolic cart was ~ 2 ml/min for VO_2_ and VCO_2_ (after controlled pure gas infusions), while in the study by Galgani et al. [16], they observed that the Vmax Encore 29n metabolic cart (SensorMedix, Cardinal Health, Germany) measurement error (after controlled pure gas infusions) reached up to ~ 40 ml/min for VO_2_ and VCO_2_. On the other hand, we should highlight that while we determined the RER, in the above-mentioned study [16], they determined the RER as the non-protein respiratory quotient (i.e., considering the urinary nitrogen excretion). Therefore, we cannot assure that other metabolic processes different than the oxidation of substrates are occurring concomitantly during the gas exchange measurements (e.g., lipogenesis) [14]. Regarding the AUC values, we observed that the AUCs (e.g., AUC RER; Table S2) provided similar results on both visits regardless of the correction of the gas exchange data procedure.

There are no doubts that an accurate and highly reproducible (i.e., reliable) metabolic cart is need to measure the gas exchange and thus, to determine the RER [16, 26]. In this regard, previous studies have shown that many of the available metabolic carts present a lack of accuracy and/or reproducibility (in both, EE and RER) [19–24]. A previous study using 6 different metabolic carts [20] showed that the day-to-day reproducibility of resting RER is better than the reproducibility of RMR. However, in general, the reproducibility of many metabolic carts is considered to be unacceptable as measurement errors of RMR ≥ 10% are not clinically admissible [34]. Therefore, in an attempt to overcome this issue, Schadewaldt et al. [25] proposed an ICcE procedure that was thought to reduce the metabolic cart error and, thus, theoretically enhance the RER and resting EE day-to-day reproducibility. In their study, Schadewaldt et al. [25] observed that after infusing known flows of N_2_ and CO_2_ into the metabolic cart’s hose tube (they used the Deltatrac [Datex Instrumentarium Co., Helsinki, Finland] and the Vmax Encore 29n systems) to simulate VO_2_ and VCO_2_ values, the values recorded by the metabolic cart deviated from the expected values in an unpredictable manner, thus rendering a “fixed correction factor” impossible [25]. These findings were confirmed later by another study [35]. However, and contrary to Schadewaldt et al.’s [25] findings, we conducted a study [36] using 4 different metabolic carts (the Q-NRG [Cosmed, Rome, Italy], the Vyntus CPX [Vyaire Medical, Höchberg, Germany], the Omnical, and the Ultima CardiO2 [Medical Graphics Co., St. Paul, MN, USA] systems), and observed that the ICcE procedure does not influence the resting RER neither the RMR day-to-day reproducibility, and that uncorrected and corrected (i.e., those obtained after applying the ICcE procedure) values were similar. It should be noted, however, that in our study [36], each gas exchange measurement lasted 30 min, and others have suggested that the ICcE procedure could be useful in postprandial assessments in which the gas exchange recordings are > 1 h [16, 26, 37].

Surprisingly, and contrary to previous suggestions [16, 26, 37], when we studied the day-to-day reproducibility of the postprandial gas exchange parameters (RER, EE, VO_2_, and VCO_2_ assessed during > 2 h), we observed that the reproducibility was similar between uncorrected and corrected values, as in our previous study [36]. Therefore, this study´s results suggest that the ICcE procedure might not be necessary when the gas exchange is measured using the Omnical metabolic cart. Nevertheless, future studies should consider our results and determine the reproducibility of their metabolic carts under both, controlled “in vitro” experimental situations (e.g., methanol burning tests), as well as under “in vivo” experimental situations (e.g., human studies) as the reproducibility of the devices may differ among different scenarios [17, 18]. Indeed, in the present study, we observed a better “in vitro” reproducibility compared to the “in vivo” during postprandial gas exchange measurements.

On the other hand, we must highlight that even using an accurate metabolic cart the day-to-day reproducibility of postprandial gas exchange cannot be considered for granted. Interestingly, the reproducibility of fasting RER and RMR in our study is comparable to those observed during 24-h assessments in a metabolic chamber [38]. We observed a CV of 2 ± 1% and of 4 ± 3% for RER and EE values (uncorrected values, RMR IC period; Table 1), while Allerton et al. [38] observed a 2% and a 4% for basal RER and EE, respectively. However, even considering the low analytical error obtained by the Omnical metabolic cart (~ 0% measurement error; Figure S1), we observed a very poor day-to-day reproducibility for the energy metabolism response to an OGTT. That is reflected in the wide lower and upper LoA (Fig. 3) and CV for the AUC RER and AUC EE. Therefore, it suggests that most of the day-to-day variability we observed is indeed attributable to the subjects (i.e., biological variability). Similar to our findings, previous studies suggested that the CV for diet-induced thermogenesis is ~ 20% [39, 40]. Therefore, taking into account the lower day-to-day reproducibility observed for the energy metabolism response, even using an accurate system as the Omnical, our results suggest that studies aiming to detect changes on MetF after an intervention would need large sample sizes. Nevertheless, we have to highlight that assessing the CV for certain gas exchange parameters (e.g., RER, AUC RER) could be problematic, as when the average of the denominator is (numerically) small, small variations are susceptible to drastically impact the CV. Therefore, a small change in the average RER could result in a large CV. Moreover, a subtle difference in the baseline could influence the subsequent postprandial response. For example, if the baseline was higher in Visit 2 than in Visit 1, the “*peak response*” would be reached sooner, and the measured postprandial response could be blunted. Importantly, we did not observe differences in our baseline values (i.e., RMR period) between days (see Table 1). In this regard, Ruddick-Collins et al. [41] studied whether the baseline RMR variability could influence the between-day variability of the postprandial response measured on 2 different days. Using a “*fixed RMR value*” (the lower of the two fasting RMR they assessed) vs. the measured baseline RMR, they observed that the between-day CV for the 6 h postprandial response was not statistically different (*P* = 0.98) between these two approaches.

Regarding capillary blood glucose concentration, we observed the expected pattern after an OGTT (i.e., an increase of glucose concentration over time), which was in agreement with the kinetics observed for the RER. Importantly, the OGTT is widely employed by both clinicians and researchers, as is more affordable and simpler than for example a euglycemic–hyperinsulinemic clamp [12]. In the present study, we did not observe statistically significant differences between days (*P* = 0.090), although our relatively small sample size might have preclude observing them after computing the AUC glucose concentration (CV ~ 11%), and we observed a good day-to-day reproducibility (CVs ranging from 3.3 to 8.3%) of the glucose concentration values measured during the different periods of the study (Fig. 1). Although we observed a good reproducibility, previous literature has suggested that OGTT day-to-day reproducibility is poor [12]. In this regard, others authors have suggested that the OGTT reproducibility is reduced in those individuals presenting a higher risk of type 2 diabetes [42–46]. Therefore, our high reproducibility may be related, in a greater or lesser extent, to the (good) health status of our participants (healthy young adults). Nevertheless, our results are in line with those observed in a recent study carried out in participants of similar characteristics (age: 24 ± 7 years; body mass index: 23.4 ± 3.6 kg/m^2^) [47]. It should be noted, however, that there is substantial variability in the blood glucose and insulin concentration during an OGTT [48]. Therefore, one could assume that the underlying metabolic processes behind these responses may influence the day-to-day reproducibility results yielded by the metabolic cart (regardless the ICcE procedure). Considering all together, the assessment of MetF—even under well-controlled and standardized conditions—may be difficult due to both, the inherent variability in the assessment test, and the accuracy of the metabolic cart used.

The present study had certain limitations. Our study was carried out in healthy young adults; thus, these findings need to be replicated in other populations (e.g., impaired glucose tolerance). As weight stability was defined by weight changes of < 3 kg, we cannot consider that all subjects were in a stable energy balance. The sample size is not too large (in part given the novelty of the study), which may impact data interpretation; thus, there is a need to test if our results replicate when a larger sample size is studied. The ICcE procedure was performed after the measurement of the participant’s gas exchange; thus, we assumed that the measurement error remains unchanged during a ~ 45-min period. We did not control the menstrual cycle of female participant [49–51], therefore, it could be possible that even considering the within-subject design and that the assessments were performed within 48 h, the hormonal variability that may be observed between cycle phases could be influencing in an unknown extent the RER or the EE day-to-day reproducibility. For logistical reasons, we determined the blood glucose concentration by a finger stick using a lancet and analyzed using a digital glucometer instead of plasma glucose intravenous samples. Moreover, we considered a steady baseline; thus, only one sample was obtained for the fasting blood glucose concentration determination. Therefore, our blood glucose concentrations values should be considered as informative values about the glucose pattern. Finally, for logistical reasons, we could not feed the participants under supervision in our research center during the preceding 24 h. Thus, participants registered the food amount in a food diary, rendering energy intake determination impossible. However, it has been suggested that several days (i.e., > 1 day) are needed to influence fasting RER [9, 52, 53], as it mostly depends on the availability of CHO at the moment of the assessment—and availability of CHO depends on the food quotient of the diet and on energy balance during the preceding days [52].

## Conclusion

Our study shows that the postprandial gas exchange parameters (AUC RER, AUC EE) assessed after two non-consecutive (48-h apart) OGTT are poorly reproducible as suggested by the observed AUCs CV (> 20%), despite using an accurate metabolic cart for the gas exchange measurement. Moreover, we did not observe any influence of the ICcE procedure on the day-to-day reproducibility, as uncorrected and corrected gas exchange parameters values were similar. Therefore, our findings suggest that the ICcE procedure is not necessary when using the Omnical metabolic cart.

## Supplementary Information

Below is the link to the electronic supplementary material.

Supplementary file1 (DOCX 519 KB)

## Data Availability

Additional data are available as on-line supplementary material. Further data are available from the corresponding author on reasonable request.
