# Supplementary material for: Reproducibility of the energy metabolism response to an oral glucose tolerance test: influence of a postcalorimetric correction procedure
Source: Eur J Nutr. 2022 Aug 25;62(1):351–61. doi: 10.1007/s00394-022-02986-w (PMC9899729; doi:10.1007/s00394-022-02986-w)
Supplement: Supplementary file 1 — Supplementary file1 (DOCX 519 KB) [file 394_2022_2986_MOESM1_ESM.docx]

**Reproducibility of the energy metabolism response to an oral glucose tolerance test: influence of a postcalorimetric correction procedure**

Alcantara JMA et al. – Online Supplementary Material

**Table S1.** Standardized meal plan options for the previous day of each testing day.

| Meal plan - Option 1 | | Meal plan - Option 2 | |
| --- | --- | --- | --- |
| *Breakfast* |  | *Breakfast* |  |
|  | Semi-skimmed milk |  | Semi-skimmed milk |
| *York ham toast* | Bread | *Serrano ham toast* | Bread |
|  | York ham |  | *Serrano* ham |
|  | Olive oil |  | Olive oil |
| *Snack* |  | *Snack* |  |
|  | Fruit |  | Fruit |
|  | Sweetened natural yogurt |  | Nuts |
| *Lunch* |  | *Lunch* |  |
| *Tomato sauce pasta* | Spaghetti or macaroni | *Tomato sauce boiled rice* | Boiled rice |
|  | Tomato sauce |  | Tomato sauce |
|  | Tuna |  | Minced pork |
|  | Olive oil |  | Olive oil |
|  | Fruit |  | Fruit |
| *Snack* |  | *Snack* |  |
|  | Fruit |  | Fruit |
|  | Nuts |  | Sweetened natural yogurt |
| *Dinner* |  | *Dinner* |  |
| *Mixed salad* | Salad | *Mixed salad* | Salad |
|  | Tomato |  | Tomato |
|  | Cheese |  | Cheese |
|  | Olive oil |  | Olive oil |
| *Baked chicken and potato* | Chicken | *Spanish omelete* | Eggs |
|  | Potato |  | Potato |
|  | Olive oil |  | Olive oil |
|  | Fruit |  | Fruit |

Participants selected one out of the two menu options and consumed it in both previous days.

**Table S2.** Area under the curve (AUC) for oxygen consumption (VO_2_), carbon dioxide production (VCO_2_), respiratory exchange ratio (RER), energy expenditure (EE), and carbohydrate oxidation (CHOox) with (corrected) and without (uncorrected) applying the individual calibration control evaluation (ICcE) procedure parameters on both testing days.

|  | Uncorrected values | | | Corrected values | | |  |
| --- | --- | --- | --- | --- | --- | --- | --- |
|  | Visit 1 | Visit 2 | Visit 1 vs. Visit 2 | Visit 1 | Visit 2 | Visit 1 vs. Visit 2 | Visit × ICcE |
|  | Mean (SD) | Mean (SD) | P | Mean (SD) | Mean (SD) | P | P |
| VO_2_ (ml/min×min) | 13 (9) | 13 (12) | 0.930 | 14 (8) | 14 (13) | 0.918 | **0.041** |
| VCO_2_ (ml/min×min) | 25 (9) | 25 (9) | 0.929 | 25 (8) | 26 (9) | 0.780 | 0.154 |
| RER | 0.06 (0.01) | 0.06 (0.02) | 0.688 | 0.06 (0.01) | 0.06 (0.02) | 0.741 | 0.973 |
| EE (kcal/day×min) | 114 (64) | 112 (84) | 0.893 | 117 (60) | 121 (84) | 0.893 | **0.036** |
| CHOox (g/min×min) | 0.07 (0.02) | 0.07 (0.02) | 0.660 | 0.07 (0.02) | 0.07 (0.02) | 0.696 | 0.956 |

Results are presented as mean and standard deviation (SD). Corrected values are these obtained after using the individual calibration control evaluation procedure. P values are from two-factor (Visit × ICcE) repeated measures analysis of variance (ANOVA, n = 12) for Day 1 and Day 2 comparisons. RER was calculated as VCO_2_/VO_2_. AUCs were expressed as ml/min×min for VO_2_ and VCO_2_, as kcal/day×min for EE, and as g/min×min for CHOox.

**Table S3.** Day-to-day reproducibility for oxygen consumption (VO_2_), carbon dioxide production (VCO_2_), and carbohydrate oxidation (CHOox) and their area under the curve (AUC) values with (corrected) and without (uncorrected) applying the individual calibration control evaluation procedure parameters at each time period.

|  | Uncorrected values | | | Corrected values | | |
| --- | --- | --- | --- | --- | --- | --- |
|  | Mean difference  (SD) | 95% LoA  (Lower ; Upper) | CV | Mean bias  (SD) | 95% LoA  (Lower ; Upper) | CV |
| Time period |  |  |  |  |  |  |
| **RMR IC** |  |  |  |  |  |  |
| VO_2_ (ml/min) | -2 (17) | (-36 ; 32) | 4.1 (2.8) | -1 (17) | (-35 ; 33) | 4.1 (2.9) |
| VCO_2_ (ml/min) | -2 (14) | (-29 ; 26) | 4.4 (2.8) | -1 (13) | (-27 ; 25) | 4.3 (2.7) |
| CHOox (g/min) | 0.01 (0.03) | (-0.06 ; 0.06) | 17.2 (15.0) | 0.01 (0.03) | (-0.06 ; 0.06) | 20.4 (19.8) |
| **1^st^ IC** |  |  |  |  |  |  |
| VO_2_ (ml/min) | -5 (18) | (-41 ; 31) | 4.4 (3.4) | -5 (19) | (-42 ; 32) | 4.5 (3.5) |
| VCO_2_ (ml/min) | -5 (13) | (-31 ; 21) | 4.1 (3.1) | -5 (13) | (-31 ; 21) | 4.2 (3.2) |
| CHOox (g/min) | -0.01 (0.03) | (-0.07 ; 0.06) | 14.6 (18.2) | -0.01 (0.03) | (-0.07 ; 0.06) | 15.8 (19.7) |
| **2^nd^ IC** |  |  |  |  |  |  |
| VO_2_ (ml/min) | -5 (10) | (-25 ; 14) | 2.5 (1.9) | -6 (11) | (-27 ; 16) | 2.5 (2.1) |
| VCO_2_ (ml/min) | -2 (8) | (-19 ; 14) | 2.4 (1.7) | -2 (7) | (-17 ; 12) | 2.1 (1.5) |
| CHOox (g/min) | 0.01 (0.04) | (-0.07 ; 0.08) | 12.3 (6.8) | 0.01 (0.04) | (-0.07 ; 0.08) | 11.7 (7.9) |
| **3^rd^ IC** |  |  |  |  |  |  |
| VO_2_ (ml/min) | 2 (39) | (-73 ; 78) | 8.3 (5.8) | 2 (39) | (-75 ; 79) | 8.5 (5.8) |
| VCO_2_ (ml/min) | 1 (37) | (-72 ; 73) | 9.9 (5.1) | 1 (37) | (-74 ; 73) | 9.6 (5.9) |
| CHOox (g/min) | -0.01 (0.07) | (-0.14 ; 0.13) | 14.9 (12.2) | -0.01 (0.07) | (-0.14 ; 0.13) | 14.3 (13.5) |
| **4^th^ IC** |  |  |  |  |  |  |
| VO_2_ (ml/min) | -3 (15) | (-33 ; 26) | 3.8 (2.4) | -4 (14) | (-31 ; 24) | 3.5 (2.5) |
| VCO_2_ (ml/min) | -2 (17) | (-35 ; 31) | 4.5 (3.5) | -3 (16) | (-33 ; 28) | 4.2 (3.3) |
| CHOox (g/min) | 0.01 (0.06) | (-0.11 ; 0.11) | 14.1 (8.4) | 0.01 (0.05) | (-0.10 ; 0.11) | 14.3 (7.2) |
| **5^th^ IC** |  |  |  |  |  |  |
| VO_2_ (ml/min) | 5 (16) | (-27 ; 37) | 3.5 (3.2) | 4 (17) | (-30 ; 38) | 3.7 (3.2) |
| VCO_2_ (ml/min) | 1 (13) | (-24 ; 25) | 3.3 (2.3) | 1 (13) | (-25 ; 25) | 3.1 (2.6) |
| CHOox (g/min) | -0.01 (0.04) | (-0.09 ; 0.06) | 8.7 (7.5) | -0.01 (0.04) | (-0.08 ; 0.06) | 8.7 (7.2) |
| **6^th^ IC** |  |  |  |  |  |  |
| VO_2_ (ml/min) | 1 (11) | (-21 ; 21) | 2.6 (1.9) | 1 (11) | (-21 ; 22) | 2.7 (2.0) |
| VCO_2_ (ml/min) | -4 (17) | (-38 ; 29) | 5.1 (3.5) | -4 (16) | (-36 ; 28) | 4.7 (3.5) |
| CHOox (g/min) | -0.02 (0.06) | (-0.13 ; 0.09) | 17.7 (14.8) | -0.02 (0.05) | (-0.13 ; 0.09) | 17.6 (14.6) |
| **AUC** |  |  |  |  |  |  |
| VO_2_ (ml/min×min) | 1 (17) | (-34 ; 35) | 88.4 (103.2) | -1 (17) | (-34 ; 33) | 75.3 (89.1) |
| VCO_2_ (ml/min×min) | 1 (13) | (-27 ; 26) | 24.6 (24.6) | -1 (13) | (-26 ; 24) | 24.7 (23.1) |
| CHOox (g/min×min) | 0.01 (0.03) | (-0.06 ; 0.05) | 19.7 (12.8) | 0.01 (0.03) | (-0.06 ; 0.06) | 23.5 (14.6) |

Results are presented as mean difference (day 1 minus day 2) and standard deviation (SD), 95% limits of agreement (LoA; lower and upper limits), and CV expressed as percentage and (SD). RMR IC is the resting metabolic rate period. Indirect calorimetry (IC) 1^st^ to 6^th^ denotes the period in which the gas exchange was recorded.

**Table S4.** Day-to-day reproducibility for glucose concentration and its area under the curve (AUC) value at each time period.

|  | Glucose concentration | | |
| --- | --- | --- | --- |
|  | Mean difference  (SD) | 95% LoA  (Lower ; Upper) | CV |
| Time period |  |  |  |
| **RMR IC** | -1 (5) | (-9 ; 9) | 3.3 (2.0) |
| **1^st^ IC** | 1 (16) | (-30 ; 33) | 8.3 (5.3) |
| **2^nd^ IC** | -1 (13) | (-26 ; 24) | 4.9 (5.7) |
| **3^rd^ IC** | 7 (10) | (-13 ; 27) | 5.5 (3.8) |
| **4^th^ IC** | 3 (13) | (-24 ; 29) | 6.1 (5.3) |
| **5^th^ IC** | 7 (9) | (-12 ; 25) | 6.4 (4.5) |
| **6^th^ IC** | 3 (13) | (-22 ; 28) | 7.7 (6.0) |
| **AUC** | 3 (5) | (-7 ; 13) | 11.1 (8.6) |

Results are presented as mean difference (day 1 minus day 2) and standard deviation (SD), 95% limits of agreement (LoA; lower and upper limits), and CV expressed as percentage and SD. Glucose concentration is expressed as milligrams per deciliter (mg/dl), while AUC glucose concentration is expressed as milligrams per deciliter per minute (mg/dl×min). RMR IC is the resting metabolic rate period. Indirect calorimetry (IC) 1st to 6th denotes the period in which the glucose concentration was determined.


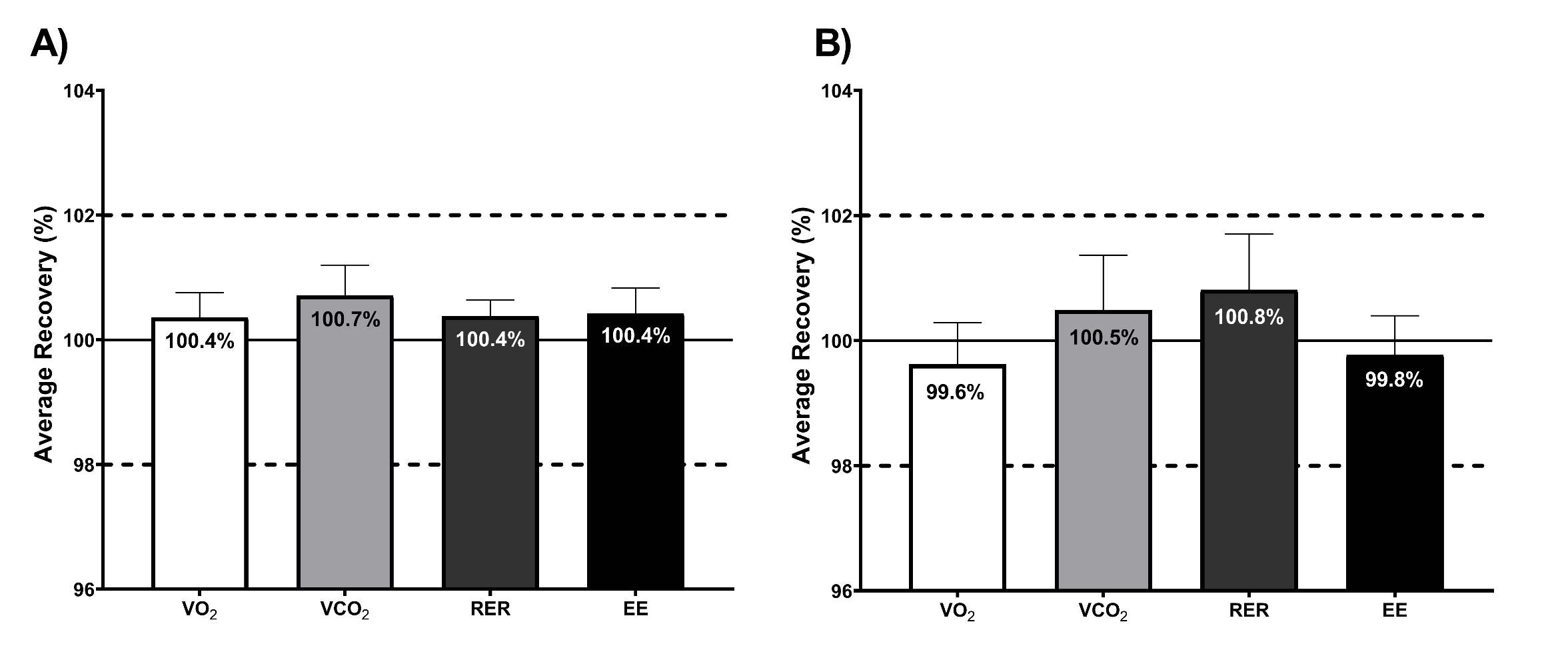
**Figure S1.** Percentage of gas exchange recovery of oxygen consumption (VO_2_; white columns), carbon dioxide production (VCO_2_; light gray columns), respiratory exchange ratio (RER; dark gray columns) and energy expenditure (EE; black columns) as determined by methanol combustion (i.e., alcohol burning test; Panel A) and by pure gas infusions (Panel B). Results (n = 9 weeks) are presented as mean and standard deviation. The expected average recovery is 100%. Dashed lines (i.e., 98% and 102%) represent the threshold that has been proposed in literature as an acceptable measurement error using these two approaches [1].


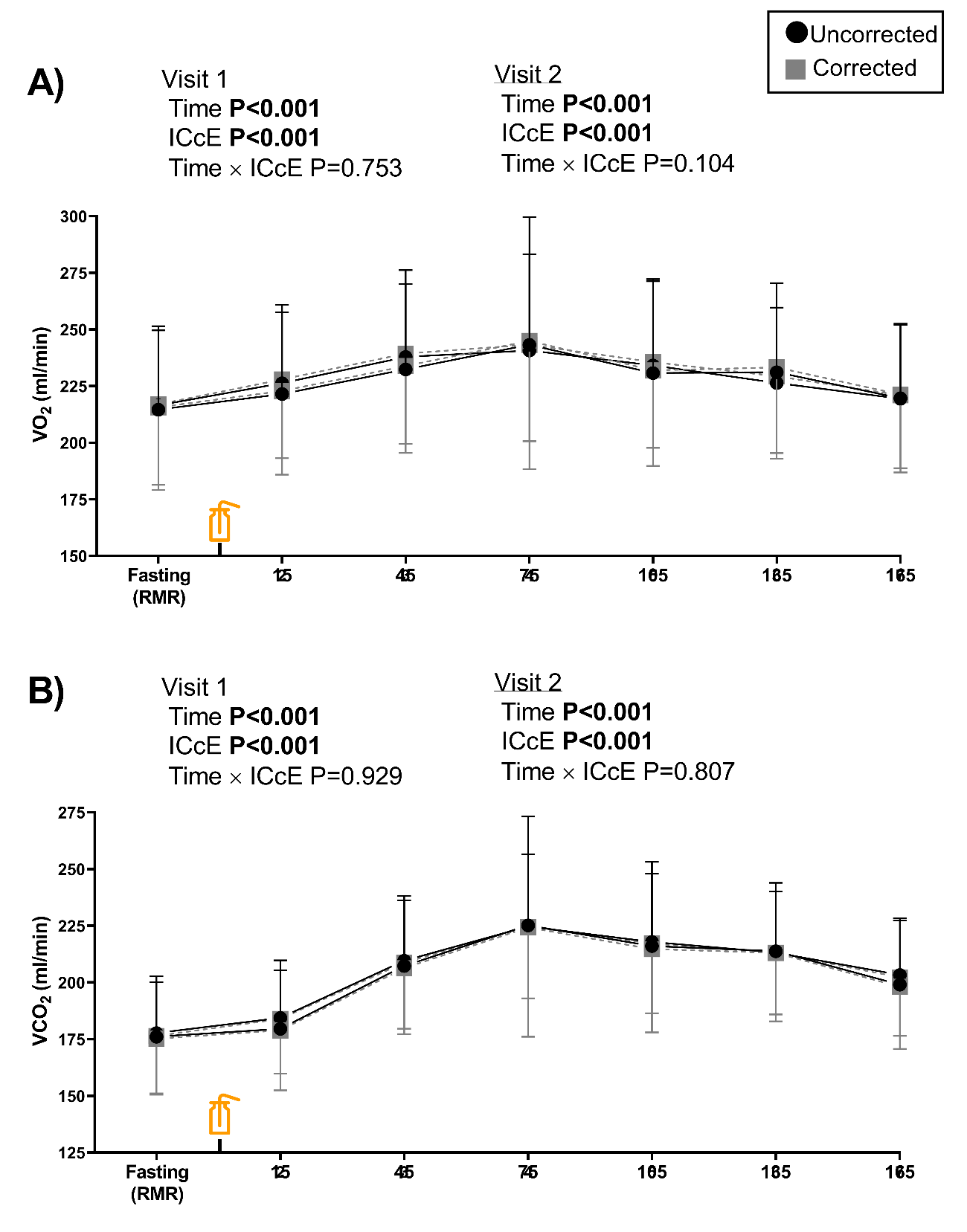


**Figure S2.** Oxygen consumption (VO_2_, Panel A) and carbon dioxide production (VCO_2_, Panel B) with (corrected values; gray) and without (uncorrected values; black) applying the individual calibration control evaluation procedure (ICcE) on Visit 1 and Visit 2 tests. Fasting RMR values correspond to the resting metabolic rate (RMR) period, i.e., before the glucose intake, while 15, 45, 75, 105, 135 and 165 represent the time in minutes after the glucose intake. The bottle icon (*x*-axis) represents the moment in which the glucose (75-g dose) was provided. Corrected values are these obtained after the ICcE procedure proposed by Schadewaldt et al. [24]. VO_2_ and VCO_2_ are expressed in milliliters per minute (ml/min). P values from two-factor (Time × ICcE) repeated measures analysis of variance (ANOVA, n = 12) for Visit 1 and Visit 2 comparisons. Results are presented as mean and standard deviation.
